# The School Medical Service

**Published:** 1920-01-10

**Authors:** 


### THE SCHOOL MEDICAL SERVICE.

### II.—Its Educative Possibilities.

The great future possibilities of the School Medical Service through the observation and treatment of the schoolchild have been considered; and it is interesting to turn attention to the opportunities which lie to hand for educative work in regard to health and general well-being. It is obviously by means of this service that the health education of the nation can and should be attempted. The Ministry of Health, which, it must be remembered, was created "to promote the health of the people," working in conjunction with the Board of Education, have it in their hands to evolve a comprehensive scheme of education in health matters which should cover the whole period of the life of the individual up to maturity. The aim of such a policy should be the creation of a demand for good health on the part of every individual. Education is and always will be criticised from many points of view, and perforce regarded as waste. To teach the individual to demand and assimilate the knowledge which will enable him to prevent disease must surely, however, be a sound principle upon which to work. No purely theoretical method of instruction is advocated; in this sphere, possibly more than in most, "example is better than precept," and reforms in housing and sanitary conditions are essential accompaniments to any scheme. Public conscience is being slowly awakened to the needs in this direction, and now is the time to press the claims of good health on the nation from all sides. No one can read Sir Geo. Newman's report and fail to be struck by the statements made by him in regard, for instance, to the condition of the teeth of the nation. We are told that the problem still remains "one of the most important, urgent, and difficult." He sees in the school service the possibilities of the foundation of a "public dental service." The time is ripe for the education of the nation in this direction, while the results of the medical examination of recruits for the Army are still in mind. Evidence is forthcoming in the report, too, that "maternal ignorance" is the cause of much unnecessary disease of all kinds.

### The Physical Condition of Entrants.

We are told in the report that there is a "serious amount of physical defect in the five-year-old child on its admission to school." This defect Sir Geo. Newman roughly subdivides into nutritional, infective, and catarrhal. Treatment is, as we saw in our last article, provided for at this stage. Much of the defect could, however, have been prevented at an earlier stage in the child's life. It is to the ante-natal clinics, schools for mothers, and infant welfare centres that we must look in the first place to remedy this state of affairs, and to give to the mother the necessary knowledge of the elementary physical needs of her infant. In our opinion, the educative aspect of this infant-welfare work is by far the most important. We use the word "educative" advisedly and in its true sense. The aim should be to foster the desire for knowledge on the part of the parent. The clinics should be used by all and sundry, "maternal ignorance" should be looked upon as a disgrace, and the ailing infant should be regarded more in the light of a reflection upon the parent and her care of it. The present figures of rickets and dental disease due to malnutrition would not then appear. One wonders sometimes whether it would not be well to cultivate in the people of our day something of the attitude of mind towards illness which is found in Butler's "Erewhon." The ideal that educational facilities as to the needs of her child should be within reach of every expectant mother and mother, and that the desire for such knowledge be present on her part, is a high one, but surely the only practical one. Schemes are already under consideration for the proper training of midwives, maternity and health visitors in larger numbers, and it is sincerely to be hoped that they will result in the formation of a large army of fully qualified and equipped workers for this important campaign.

### The Health Education of the Child.

Sir George Newman lays stress upon the importance of the healthful environment of the Public Elementary School as an educative factor in the life of a child. When he suggests that the fundamental subject of hygiene should be taught in all schools "as a way of life and habit rather than in the abstract form of a body of theoretical information," he strikes a note which we would echo to the full. As he says, "it is in the elementary, secondary, and continuation schools that there must be laid the foundation of the public health." We would recommend that the teaching of the child be begun at an even earlier age, the opportunities will be to hand in the nursery school. Experience has shown that habits of cleanliness can be inculcated at a very early age. If the care of the health were carefully interwoven in the general scheme of education, and not treated as a separate and distinct subject, untold benefit might result. The problems in connection with instruction on sex-hygiene would solve themselves, if children during their school life had come to regard personal health and well-being as part of their normal education.

### Physical Instruction in the Schools.

The encouragement of a love of healthy physical exercise and suitable recreation is one of the most valuable means of health education in the schools. With the Education Act comes the power of providing physical education and recreation for all children and young people up to eighteen years of age. Sir George Newman considers that if a full and effective organisation can be established a great reform will be introduced in the daily life of the

The Previous article appeared Dec. 27, p. 283.

**The School Medical Service—(*continued*).**

community, and in urging its claims upon local authorities, he deplores the fact that physical education is not yet by any means performing the function it should in our educational scheme. Camps, and play centres, as well as drill, dancing, swimming, organised games, etc., find their place in this instruction. Public schools have emphasised this side of education for its physical and especially its character-forming value. It is now possible for the elementary, secondary, and continuation schools to devise some adequate and comprehensive scheme to be carried out by organisers and teachers specially trained to realise the educative possibilities of the work. There are difficulties to contend with, but it should be possible to enlist enough public sympathy in the matter to ensure the acquisition in large towns, for example, of more open spaces and swimming baths.

### The Feeding of the Schoolchild.

The policy of the feeding of the schoolchild would seem to be essentially a changing one, in view not only of recent research as to the essentials of dietary in the form of vitamines or accessory food factors, but also in view of fluctuations in industry and rates of wages. Wherever such feeding is carried on by a public body, however, we think it might not be straining a point to regard it as having some potential educational value. Naturally the proper feeding of the child is the duty of the parent, but at the present time any meals given to children which tend to cultivate a taste for suitable food will be of some value. In a successful health campaign the nation will have to learn what to eat, and the taste of the children must be educated.

### Health and the Juvenile in Industry.

The education of the child in health matters having been begun in infancy, and continued through school life, can be carried on into the early years of employment, which are the critical years of adolescence. On the foundation which has been laid during school life can be built a structure having far-reaching results, moral and physical. Reference is made in the Report to some work carried out recently in London at some of the juvenile unemployment centres formed after the signing of the Armistice, at which one of the features was a health talk by the examining doctor, which proved to be of considerable value. The foundations of the interest in the advice given had doubtless been laid by the visits of the school doctor in earlier days.

The work begun in the schools is not going to be undone or stopped in the works. One of the most hopeful signs of the present day is the interest in the physical welfare of the worker which is being aroused in the industrial world. The true relation between output and the physical well-being of the worker is being realised. The Report comments on the clauses in the constitution of the Joint Industrial Councils (Whitley Councils) relating to the improvement of health conditions in the industry and to medical treatment. "The aim is to secure a health conscience in employer and employee." It is the work of the School Medical Service in its widest sphere in great part to form this conscience, besides making available the medical records of the juvenile if required by the certifying surgeon or examining doctor. Much co-operation is necessary between sanitary authorities, welfare workers, factory inspectors, and employers, but we cannot but believe that this will come if the worker has a real desire, first and foremost, for cleanliness in person and surroundings, then for healthy recreation of all kinds, for proper ventilation, and for facilities for inspection and treatment. If the School Medical Service, taking full advantage of its extended powers, creates a "health conscience" and an understanding of the meaning of good health in the coming generation, it will have performed a task of untold value to the nation. The outlook is full of hope. Admittedly the task is formidable, and its successful carrying out presupposes a full realisation of the ends to be aimed at on the part of teachers and others. No new scheme either of medical work or education is needed, however; all is there, but the need is for a fuller, wider, and more comprehensive use of facilities already available by means of the new powers and duties conferred by recent legislation from the point of view of national health and well-being.

